# Photoinduced Oxidation of Lipid Membranes in the Presence of the Nonsteroidal Anti-Inflammatory Drug Ketoprofen

**DOI:** 10.3390/membranes12030251

**Published:** 2022-02-22

**Authors:** Anna V. Mastova, Olga Yu. Selyutina, Veronika I. Evseenko, Nikolay E. Polyakov

**Affiliations:** 1 Institute of Chemical Kinetics and Combustion, Institutskaya St. 3, 630090 Novosibirsk, Russia; mastova-anna99@yandex.ru (A.V.M.); polyakov@kinetics.nsc.ru (N.E.P.); 2 Institute of Solid-State Chemistry and Mechanochemistry, Kutateladze St. 18, 630128 Novosibirsk, Russia; evseenko@solid.nsc.ru

**Keywords:** lipid membranes, ketoprofen, photosensitivity, phototoxicity, free radicals, decarboxylation, radical polymerization, CIDNP, molecular dynamics

## Abstract

The damage of cell membranes induced by photosensitive drugs has attracted the significant attention of researchers in various fields of medicine. Ketoprofen (KP) is known to be the most photosensitive among the nonsteroidal anti-inflammatory drugs. The phototoxic side effects of KP and other non-steroidal anti-inflammatory drugs are associated with the action of free radicals, but there is insufficient information about the nature of these radicals. In the present study, free radicals formed upon KP irradiation within lipid membranes were studied using nuclear magnetic resonance (NMR) and chemically induced dynamic nuclear polarization (CIDNP) methods, as well as a molecular dynamics simulation. Our study confirmed the effective penetration of KP into the lipid bilayer and showed a significant effect of the nature of the medium on the photolysis mechanism. While, in a homogeneous solution, the main channel of KP photolysis is free radical-mediated monomolecular decomposition with formation of radical pairs of benzyl and CO_2_H^●^ radicals, then, in the lipid membrane, the reaction route shifts towards the bimolecular reaction of KP photoreduction. In addition, the effect of the presence an electron donor (the amino acid tryptophan) on lipid oxidation has been studied. It was found that photoreaction of KP with tryptophan proceeds more efficiently than with lipid molecules.

## 1. Introduction

The interaction of photosensitive drugs with cell membranes has attracted significant attention of researchers in various fields of medicine. Membranes provide stable cell functioning and transport of small molecules and ions between the cell and its environment. Also, it fixes the position of membrane-associated proteins and influences their structure and activity. The lipid bilayer constitutes the basis of the cell membrane. The damage of cell membranes caused by photoinduced oxidation of lipid molecules and membrane-associated peptides may induce various phototoxic and photoallergic reactions. These reactions are described in a number of reviews and research articles [1,2,3].

**Figure 1:**
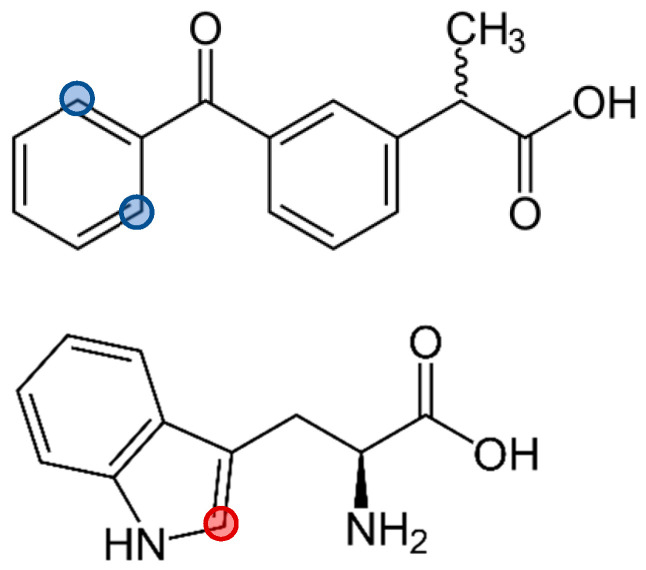
The structure of ketoprofen (KP, 2-(3-benzoylphenyl) propanoic acid, **top**), and tryptophan (**bottom**).

The nonsteroidal anti-inflammatory drug ketoprofen (KP, 2-(3-Benzoylphenyl) propanoic acid) causes photo-contact dermatitis under ultraviolet light as a side effect [3,4,5,6,7,8]. Also, it is known that its photoproducts may induce photoallergic reactions [9,10]. Due to the presence of a benzophenone chromophore in KP structures (Figure 1), it can participate in various photo–redox processes with the formation of toxic free radicals and potentially allergic photoproducts. The set of KP photoproducts has been described earlier in a number of studies performed in different media, in both aerobic and anaerobic conditions [11,12,13]. It was established that the set of ketoprofen photoproducts differs substantially from that typical for benzophenone, emphasizing the differences in the mechanisms of photodegradation of these two ketones. It should be noted that the molecular mechanisms of KP photo-transformation, and especially the nature of short-lived paramagnetic intermediates of KP, are still under debates [12,14,15,16,17].

In particular, S. Babenko with co-authors, using chemically induced dynamic nuclear polarization (CIDNP) and nuclear magnetic resonance (NMR) techniques, have discovered a new radical photo-transformation channel during ketoprofen photolysis in a homogeneous acetonitrile solution [16]. This mechanism includes the breaking of the C-C bond in the triplet excited state of KP with the formation of the neutral radical pair of benzyl and CO_2_H^●^ radicals, in contrast to the earlier suggested elimination of neutral CO_2_ molecules under photolysis [11,12]. In addition, the authors of [16] showed that in the presence of hydrogen donors, an additional radical reaction channel, namely, photoreduction with the formation of a ketyl radical, can occur. The competition between these reaction channels depends on the nature of the environment and on the presence of a suitable hydrogen donor.

In the cell membranes, such hydrogen donors might be lipid molecules and various amino acid residues. The role of amino acids in KP photo-transformation in homogeneous solution was studied in detail by T. Suzuki, with coauthors using transient absorption techniques and quantum chemical calculations [18,19,20,21]. It was found that the amino acids tryptophan, tyrosine, and basic amino acids such as histidine can accelerate the reaction rate of KP decarboxylation to produce the biradical in the phosphate buffer solution. In addition, the transient absorption data indicate that triplet excited state of KP abstracts a hydrogen atom of the N−H group in the indole frame of tryptophan [21]. These findings give authors the information on the reactivity of excited KP in the vicinity of tryptophan in a KP−protein complex, which can cause photosensitization in human skin.

The membrane damage by ketoprofen in vitro was detected during the photolysis of red blood cells and cultured hepatocytes and fibroblasts [22]. A recent study of S. Okazaki with co-authors have attempted to detect the lipid-derived radicals using electron paramagnetic resonance (EPR) techniques [15]. The authors examined radical reactions induced by ketoprofen in the lipid membranes consisting of egg yolk phosphatidylcholine labeled with a stable nitroxyl radical (NR) at the 5- or 16-position of the fatty acid chain, respectively. When the suspension of liposomes mixed with ketoprofen was irradiated by UV light, the EPR signal from NR decreased due to reaction with free radicals produced by KP. The overall decay for NR at the fifth position was faster than that for the sixteenth position. This result indicates that ketoprofen penetrates into the lipid membrane and induces a radical reaction near the polar region in the membrane. In addition, an EPR spin trapping study allowed researchers to detect the C-centered radical adduct after UV irradiation. However, the origin of this C-centered radical was not established.

Taking into account the important role of free radicals in the mechanism of ketoprofen phototoxicity, in this work we have studied the photolysis of KP in a phospholipid bilayer that mimics the biological environment, and tried to trace how the nature of the medium affects the efficiency and mechanism of KP photodecomposition. The interaction of KP with the lipid bilayer and tryptophan molecules was investigated by ^1^H NMR techniques and molecular dynamic (MD) simulations at different pH. The short-lived paramagnetic intermediates formed during photoinduced interaction of ketoprofen with lipid molecules and amino acid tryptophan have been studied using a chemically induced dynamic nuclear polarization technique.

## 2. Materials and Methods

### 2.1. Materials

**Figure 2:**
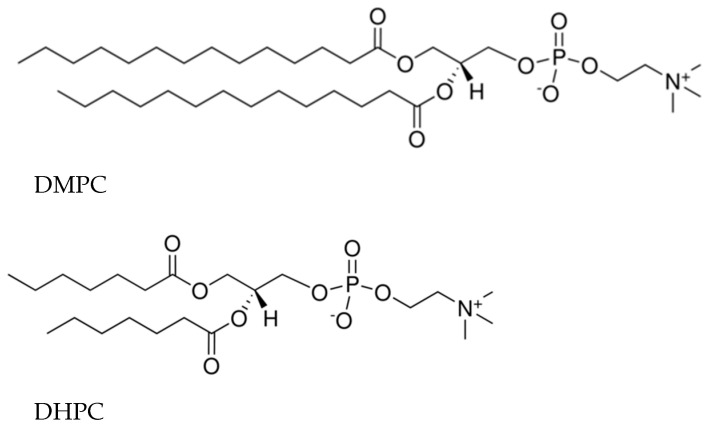
Structures of lipids DMPC (1,2-dimyristoyl-sn-glycero-3-phosphocholine) and DHPC (1,2-diheptanoyl-sn-glycero-3-phosphocholine).

S-Ketoprofen (98%, Sigma-Aldrich, St. Louis, MO, USA), L-tryptophan (Trp, 98%, Sigma-Aldrich, St. Louis, MO, USA), and deuterated solvent D_2_O (99.9%D) were used as received. Bicelles were formed from DMPC (1,2-dimyristoyl-sn-glycero-3-phosphocholine) and DHPC (1,2-diheptanoyl-sn-glycero-3-phosphocholine, Avanti Polar Lipids, Alabaster, USA, purity > 99%, Figure 2). Powdered components (lipids, KP, Trp) were dissolved in chloroform, the solvent was dried, and the resulting film was hydrated with phosphate buffered saline (PBS) or water. Necessary amounts of DCl or KOD were added to reach the required pH value. To accelerate the formation of bicelles, three freeze–thaw cycles were performed. The DMPC: DHPC ratio was 1:2, with the total lipid concentration being 24 mM.

### 2.2. NMR and CIDNP Study

^1^H NMR and selective NOESY spectra were obtained on a Bruker (Billerica, MA, USA) Avance HD III NMR spectrometer (500 MHz ^1^H operating frequency). T_1_ relaxation times were obtained using standard inversion–recovery pulse sequence. CIDNP experiments were performed on a Bruker DPX-200 NMR spectrometer (200 MHz ^1^H operating frequency) equipped with a Lambda PhysikEMG 101 MSC (Göttingen, Germany) excimer laser, used as a light source (308 nm, 100 mJ at output window, 20 mJ/pulse in sample volume, pulse duration 15 ns) in the CIDNP experiments. The samples were irradiated in standard 5 mm Pyrex NMR tubes (Corning, New York, USA) directly in the NMR probe of the spectrometer. The samples were bubbled with argon for 15 min to remove dissolved oxygen prior the experiment. To enhance the signal-to-noise ratio in the present study, we used the pseudo steady-state (PSS) photo-CIDNP method. The PSS experiments were performed using standard pulse sequence: presaturation—delay 1—pulse τ(π)—delay 2 (16 laser flashes with repetition rate 50 Hz during delay 2)—observation pulse τ(π/2)—acquisition. Delay 1/delay 2 = 1.1 to remove residual signals of solvents and solutes. Before and after irradiation. the ^1^H NMR spectra of the reaction products were recorded on an Avance HD III NMR spectrometer.

### 2.3. Molecular Dynamics Simulations

Molecular dynamics simulations were performed to understand the interactions of KP with phospholipid-containing membranes and Trp molecules using the Gromacs 2018.4 package and GROMOS54a7 force field. The topologies of KP and Trp were built using the Automated Topology Builder [23]. For lipid simulations, the model lipid DMPC (1,2-dimyristoyl-sn-glycero-3-phosphocholine) introduced by Poger and Mark was utilized [24]. The simple point charge (SPC) model of water molecules was used.

The simulation was performed in the isothermal–isobaric (NPT) ensemble with constant pressure (1 bar) and constant temperature T = 310 K, which were maintained by the semi-isotropic Parrinello–Rahman barostat [25] and Nose–Hoover thermostat [26]. For electrostatic interactions, the particle mesh Ewald (PME) method with the fourth-order of cubic interpolation and a grid of 0.16 was used [27]. The initial configuration of the system contained the bilayer, consisting of 128 lipid molecules surrounded by water (~10,000 water molecules) and KP and Trp molecules located in water outside the bilayer. For all systems with KP and Trp, one production run of 500 ns duration was performed.

## 3. Results

### 3.1. KP Interaction with Lipid Bilayer Studied by NMR Technique and Molecular Dynamic Simulation

**Figure 3:**
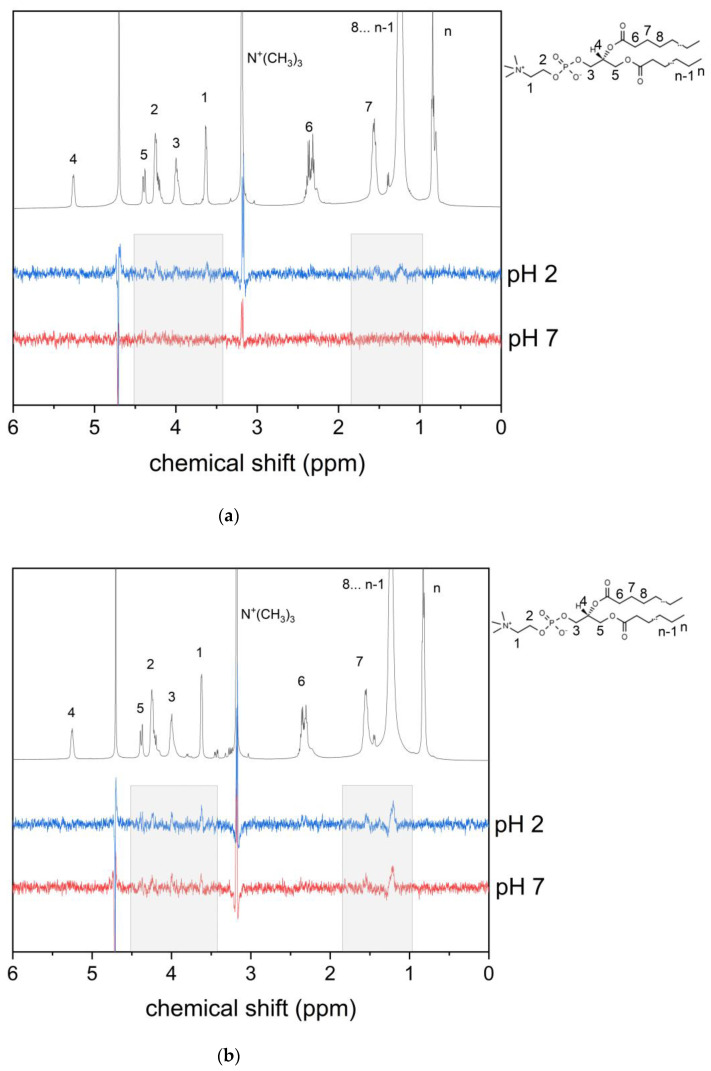

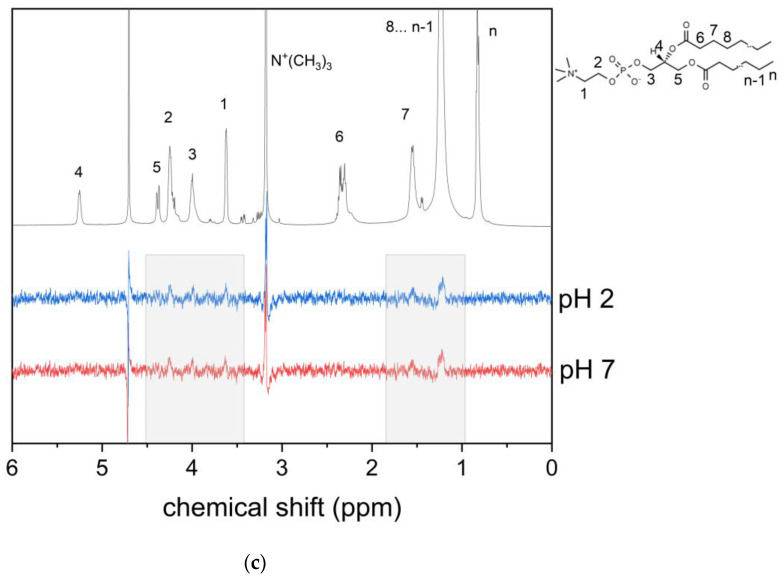
1D NOESY spectra (blue and red lines) and ^1^H NMR spectra (gray lines) of (**a**) 2 mM KP; (**b**) 2 mM KP + 4 mM Trp, KP protons; (**c**) 2 mM KP + 4 mM Trp, Trp protons in DHPC/DMPC bicelles at different pH values. Mixing time was 500 ms. Selective excitation of KP protons at 7.8 ppm (Figure 1, blue circles) and Trp protons at 7.2 ppm (Figure 1, red circle) was performed.

At the first stage, we applied the NMR technique and molecular dynamic simulation to study the interaction of KP molecules, in protonated and deprotonated forms, with lipid bilayer and amino acid tryptophan. Selective 1D NOESY spectra of KP in bicelles at different pH values are given on Figure 3. Selective excitation of KP protons at 7.8 ppm (Figure 1, blue circles) was performed.

Cross-peaks of the signals of KP (chemical shift 7.8 ppm, blue circles at Figure 1) with phospholipid signals of N^+^(CH_3_)_3_-groups were observed in all cases. In the acidic media, small cross-peaks of acyl CH_2_- at 1.2 ppm and from CH_2_-groups one, two, and three were also observed (Figure 3, gray rectangles). It is worth noting that the pKa value of KP is 4.7 [18]. In the absence of Trp, these peaks disappear under KP deprotonation (pH = 7, Figure 3a), but in the presence of Trp, these peaks are independent from pH (Figure 3b). Cross-peaks at NOESY spectra were observed when the distance between the nuclei was less than 0.5 nm. Obtained results mean that, firstly, the KP molecule is able to penetrate into the lipid bilayer, and secondly, the KP–bilayer interaction is sensitive to the presence of Trp.

The same cross-peaks of Trp protons (chemical shift 7.2 ppm, red circles, Figure 1) were observed (Figure 3c). Obtained results indicate that the Trp molecule is able to penetrate into the lipid bilayer. We have also determined KP and Trp T_1_ relaxation times in water solution and in bicelles in media with different pH, in the presence and in the absence of tryptophan molecules (Table 1).

It is notable that the spin–lattice relaxation times of ketoprofen protons decreases by approximately half in bicelles in comparison to water solution. NMR relaxation times are very sensitive to molecular motion. Limitation of the mobility of the molecule leads to a decrease in the relaxation time. The approach based on determination of NMR relaxation times is widely used to distinguish whether either molecule is in a free or bounded (supramolecular complex, dimer, membrane-bounded) state [28,29,30,31,32,33]. At the same time, in the presence of Trp, the T_1_ of KP in the deprotonated state (pH = 7) is the same as in pure water. However, as it was mentioned above, NOESY cross-peaks of KP protons with lipid acyl chain signals were still observed. Probably, KP is still able to penetrate into lipid bilayer, but the exchange between bilayer and water takes place. The same results are obtained for the Trp molecule.

**Figure 4:**
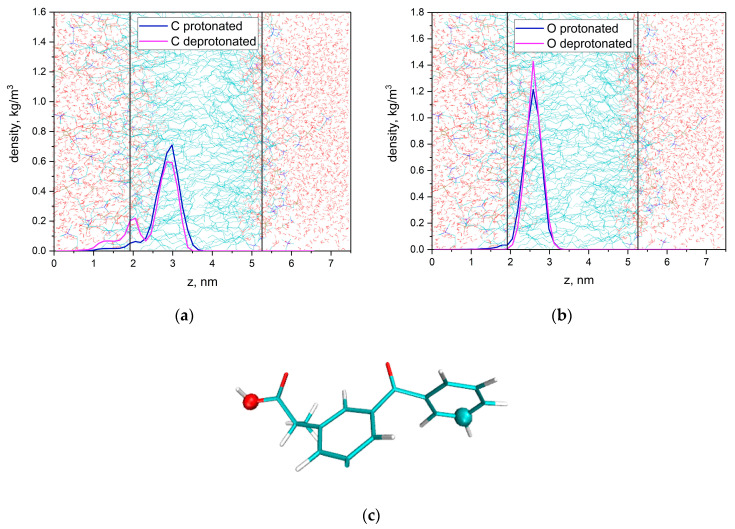
Density profiles of the selected C (**a**) and O (**b**) atoms of ketoprofen in protonated and deprotonated states and atom selections (**c**). Vertical lines correspond to the centers of density profiles of DMPC N atoms.

These experimental results were confirmed by MD simulations. Figure 4 shows calculated density profiles of the selected C and O atoms (see Figure 4c) across the box. The lipid bilayer is centered at the center of the box. The protonated KP molecule quickly (~20 ns) penetrates into lipid bilayer and is located near lipid headgroups in the hydrophobic part of the bilayer. The deprotonated KP molecule also quickly (~20 ns) penetrates into the lipid bilayer, but in contrast to the protonated molecule it could leave the hydrophobic region, remaining bound to the bilayer surface. Obtained results are in good agreement with the results of Okazaki et al. [15], which indicate that the KP molecule penetrates into the lipid bilayer.

**Figure 5:**
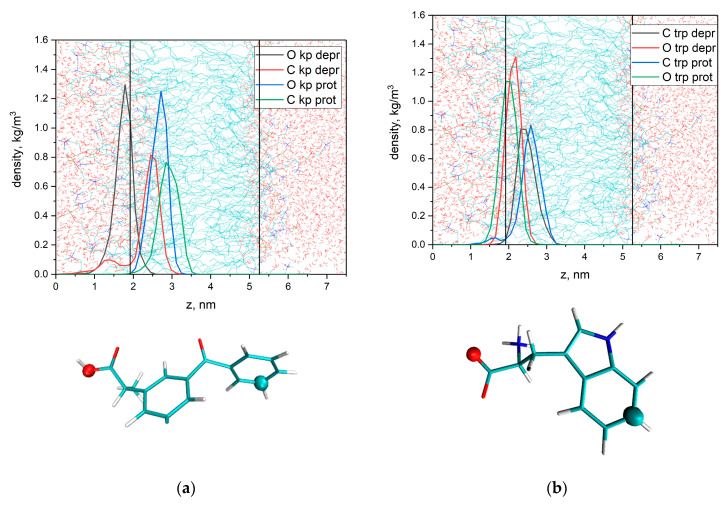
Density profiles of the selected C and O atoms of ketoprofen (**a**) and tryptophan (**b**) in the protonated and deprotonated ketoprofen state. Vertical lines correspond to the centers of density profiles of DMPC N atoms.

Density profiles are slightly different in the presence of Trp molecule (Figure 5). In the deprotonated state, the KP molecule is located significantly closer to the surface of the lipid bilayer than in the absence of Trp. We have also calculated the mean distances between selected C atoms of KP and Trp (see Figure 5) from MD trajectories. For the protonated form of KP, the mean distance is 1.6 ± 0.5 nm; for the deprotonated form, the mean distance is 3.2 ± 0.3 nm. So, in the case of deprotonated KP molecule, the mean distance between aromatic protons of KP and Trp is two times higher than in the case of protonated KP molecule.

**Figure 6:**
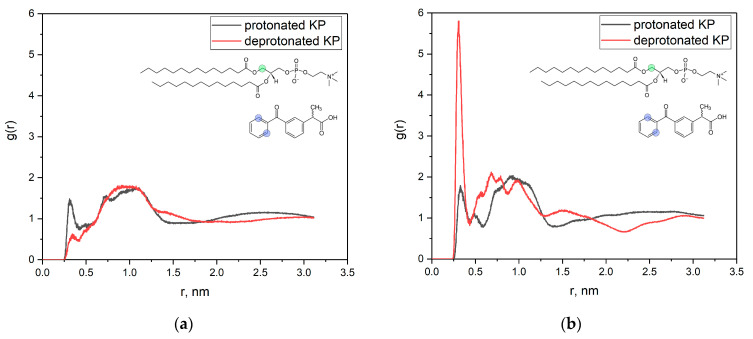
Radial distribution function g(r) of the hydrogens of KP (blue circles) and CH_2_-groups of DMPC (green circle) (**a**) in the absence of tryptophan; (**b**) in the presence of tryptophan.

Different behavior of KP molecule in the bilayer in the absence and in the presence of tryptophan could be the reason for the observed differences in selective NOESY experiments. We have calculated the radial distribution functions (g(r)) of KP hydrogens and DMPC CH_2_-groups (Figure 6) for protons corresponding to cross-peaks observed in NOESY experiments. It could be seen from Figure 6 that, in the absence of Trp g(r), the selected atoms for the distances of less than 0.5 nm, are much less for deprotonated KP.

It means that the probability of these atoms to have a distance less than 0.5 is much less for deprotonated KP than for the protonated one. In contrast, in the presence of Trp, this probability is significantly higher for deprotonated KP (Figure 6b). As was mentioned above, NOESY signals were observed for protons at the distance of less than 0.5 nm. Probably, the low probability of the selected atoms to be at this distance leads to a decrease in the number of protons capable of producing a NOESY signal below the sensitivity limit. In the presence of Trp, this probability is high and NOESY signals were observed.

Thus, NMR experiments and MD simulations revealed that the KP molecule could interact with the lipid bilayer in both its protonated and deprotonated form; however, the depth of KP penetration into bilayer is different in media with different pH. Also, KP localization is sensitive to the presence of tryptophan molecules in the system.

### 3.2. KP Photolysis in Phospholipid Bicelles

**Figure 7:**
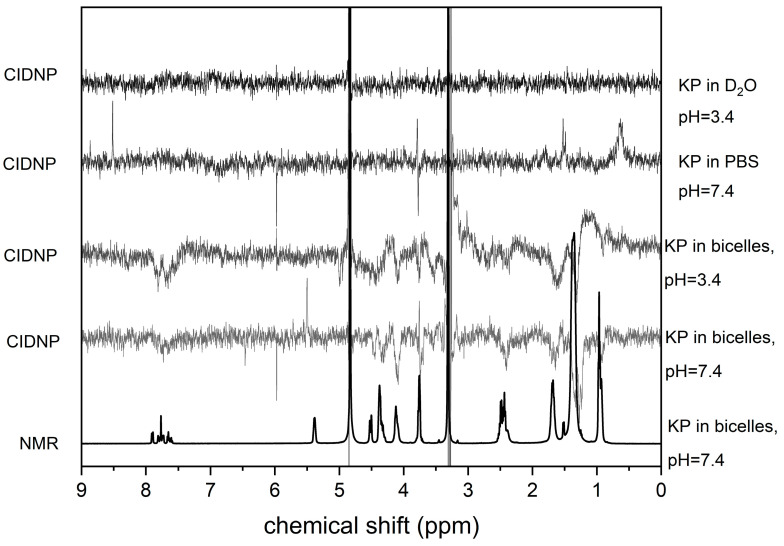
^1^H NMR and CIDNP spectra of KP in water solution and DHPC/DMPC bicelles at different pH of the media.

In the present study the CIDNP method has been applied to study the radical intermediates formed in the photolysis of KP in lipid bilayers and the observed effects were compared with the CIDNP effects detected under KP photolysis in homogeneous solution. CIDNP phenomenon is the appearance of a non-equilibrium distribution of the intensities of NMR signals during chemical reactions proceeding with participation of free radicals. Note that CIDNP effects are formed in the pair of two radicals formed via breaking the chemical bond or via electron or hydrogen atom transfer between a corresponding donor and acceptor, and are the direct evidence of the involvement of free radicals in the reaction scheme [34]. Moreover, taking into account that the CIDNP intensity is proportional to the values of hyperfine interaction constants in the radical precursor of polarized products, the CIDNP spectrum can be considered as a portrait of all free radicals participating in the reaction [35]. Figure 7 shows the CIDNP effects detected in the photolysis of KP in PBS and in lipid bilayer.

**Scheme 1:**
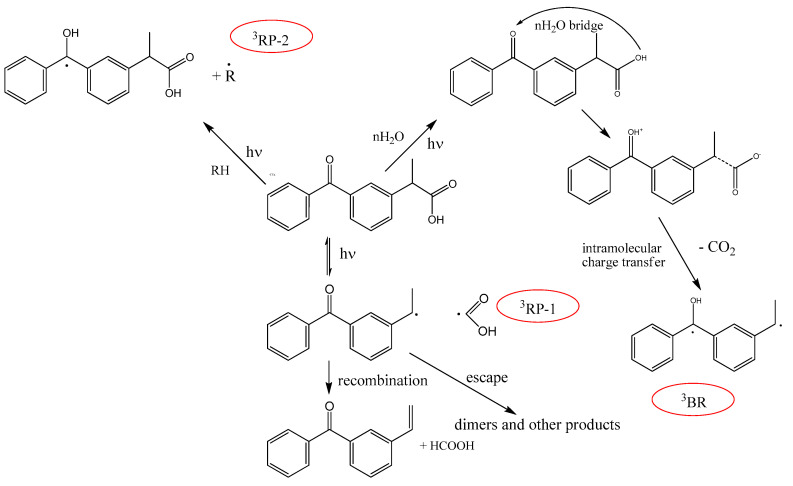
Suggested reaction channels of KP photodegradation.

The CIDNP effects detected during KP photolysis in homogeneous acetonitrile have been described earlier [16]. Polarization of initial KP leads to the enhanced absorption (A) at CH_3_ group (1.4 ppm) and emission (E) at CH group (3.8 ppm), but the absence of polarized aromatic protons was detected. It means that in acetonitrile solution, the main photodegradation channel is monomolecular decay, with breaking C-C bonds and the elimination of ^●^COOH radicals. This reaction is reversible, and polarization is formed in triplet radical pairs of benzyl and ^●^COOH radicals (^3^RP-1, Scheme 1). Recombination of this radical pair gives the polarized KP molecule. In addition, the polarized formic acid (A, 8.1 ppm), formed by disproportionality of benzyl and ^●^COOH radicals has been observed. The CIDNP formation in this radical pair was described in detail earlier [16].

In aqueous solutions, the CIDNP intensity decreased significantly. We can assume that the decrease in CIDNP intensity in aqueous solution was due to increase of intramolecular electron and proton transfer channels in the triplet excited state of KP, triggered by water molecules. According to literature data, in aqueous solutions, the lifetime of the excited triplet state of KP is dramatically shorter than that obtained in neat acetonitrile solution [36]. Using time-resolved resonance Raman spectroscopy, Li with co-authors have shown that water molecules may act as bridges to mediate intramolecular proton transfer in the triplet excited state of KP and subsequently generate a triplet-protonated carbanion biradical species (3BR, Scheme 1) [17].

Although water is considered to be a relatively “inert” hydrogen donor solvent, the DFT calculations show that the activation energy barrier for the triplet-state intramolecular proton transfer and associated decarboxylation process become lower when water molecules are involved in the reaction system. In PBS, polarized products of the monomolecular decay of KP (HCOOH, 8.5 ppm), as well as polarization on the CH_3_ group of KP (1.5 ppm, Figure 6) were still observed. In an acidic medium (pH 3.4), the effects of CIDNP were not observed, possibly due to the low solubility of KP. Another possible reason of the absence of polarization in acidic media is further acceleration of intramolecular processes in the triplet excited state of KP. Acid can shuttle a proton from the carboxyl to carbonyl group through an initial intramolecular proton transfer, which facilitates the cleavage of the C-C bond, thus leading to the decarboxylation reaction.

In contrast to PBS, KP is high soluble in bicelles suspension, due to inclusion in bicelles. There are no characteristic signals of the reaction of monomolecular decomposition of KP in the NMR and CIDNP spectra after photolysis, which indicates that KP interactions with lipid molecules is the main reaction channel. We can assume that water molecules are not involved in the reaction when KP is located inside the lipid bilayer. The CIDNP intensity in this reaction is very weak, the polarized signals of lipid protons are observed, as well as of aromatic protons of KP or its phototransformation products (7.5–8 ppm). The observation of polarized lipid protons is the evidence of lipid radical formation in this reaction.

### 3.3. KP Photolysis in Phospholipid Bicelles in the Presence of Electron Donor Tryptophan

As was mentioned in the Introduction, real cell membranes contain various amino acid residues. Transient absorption techniques and quantum chemical calculations show that the amino acids tryptophan, tyrosine, and basic amino acids such as histidine can accelerate the reaction rate of KP decarboxylation to produce biradicals in the homogeneous phosphate buffer solution [18,19,20,21]. In addition, the transient absorption data indicate that the triplet excited state of KP abstracts a hydrogen atom of the N−H group in the indole frame of tryptophan [21]. Using the CIDNP technique, it was demonstrated that the first step of this reaction is electron transfer [37]. The possibility of electron transfer between KP and Trp molecules under light irradiation has been confirmed in our previous study [37]. According the Rehm–Weller–Zachariasse criterion, the processes of electron transfer will be favorable if the free energy changes are negative [38].

**Figure 8:**
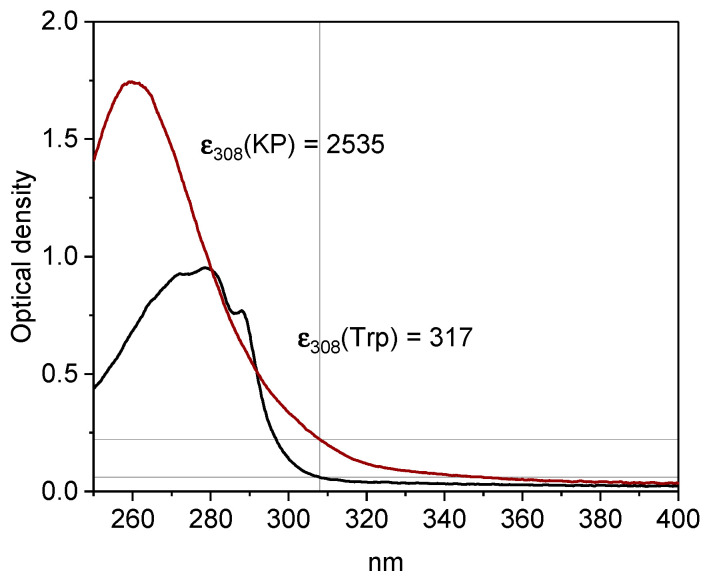
Absorption spectra of KP (0.1 mM) and Trp (0.2 mM) in bicelles at pH 7.4. Extinction coefficients are shown for wavelength of laser light, 308 nm.

In the NMR experiments performed in the present study, when Trp was added to KP, located in lipid bilayer, a shift of the KP lines was observed in the NMR spectrum, which indicates their interaction within the bilayer. This suggestion was confirmed by molecular dynamics simulation (see Section 3.1). As it was shown by molecular modeling, the protonated form of KP is closely located to Trp molecule inside membrane, but the deprotonated form of KP is expelled by tryptophan from the lipid bilayer. The further study demonstrated that such behavior has a strong influence on the photochemical processes in the membrane. In the presence of tryptophan, the rate of KP decomposition decreases, which may be due to the reversibility of the electron transfer reaction from tryptophan to the KP triplet. Note that light in this reaction is predominantly absorbed by KP molecules, whose extinction coefficient is an order of magnitude higher than that for Trp. The absorption spectra of KP and Trp in bicelles are shown in Figure 8.

**Figure 9:**
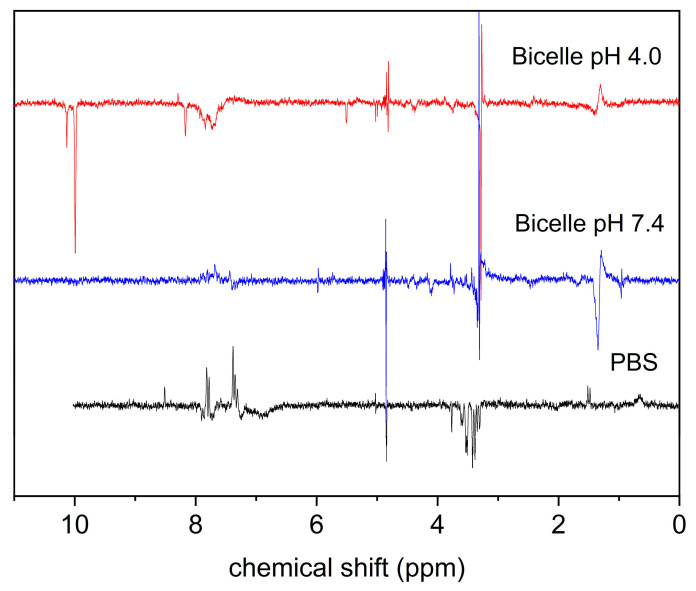
CIDNP spectra detected during photolysis of a mixture of KP (2 mM) + tryptophan (4 mM) in phosphate buffer and DMPC/DHPC bicelles at various pH (aerated solution).

Figure 9 shows CIDNP spectra detected during photolysis of KP in the presence of Trp in PBS buffer and in the lipid bilayer at different pH values of solution. In the absence of bicelles, the presence of CIDNP effects on both the tryptophan protons and on the reaction products indicate that the reaction of electron (or hydrogen atom) transfer in the photoinduced interaction of KP with Trp takes place (Figure 9).

**Figure 10:**
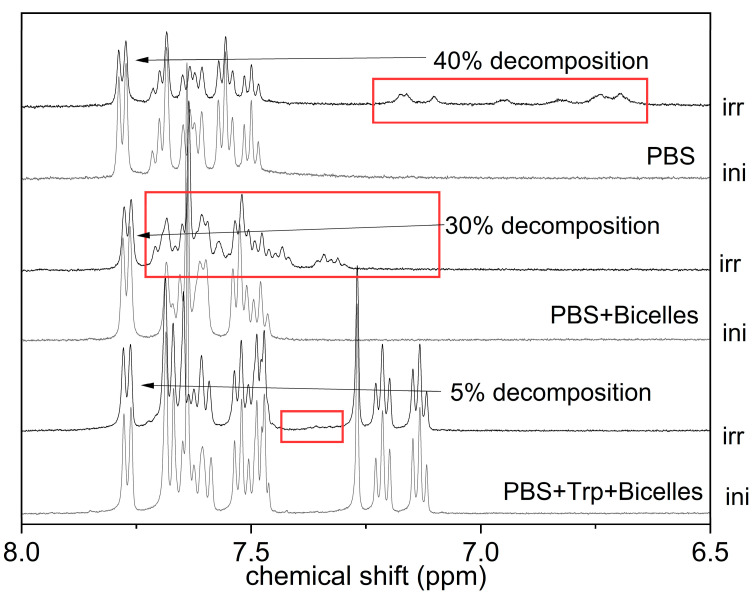
NMR spectra (fragments) of KP solutions in aerated PBS (pH 7.4) before (ini) and after photolysis (irr, 64 laser pulses). Concentrations of KP = 2 mM, Trp = 4 mM. The reaction products are marked with red rectangles.

CIDNP signs of Trp protons (A on aromatic protons, 7.4 and 7.8 ppm and E no CH_2_ group, 3.4 ppm) correspond to triplet radical pair (a(indol) < 0, a(CH_2_) > 0, Δg < 0). In PBS, polarized products of the monomolecular decay of KP (HCOOH, 8.5 ppm), as well as polarization of the CH_3_ group of KP (1.5 ppm) were still observed. In bicelles, there is no CIDNP on aliphatic protons of KP, which, together with the absence of the corresponding products of monomolecular decomposition, indicates that the channel of the monomolecular decomposition is minor in the photolysis of KP in the lipid bilayer. This is also indicated by a different set of photolysis products in homogeneous solution and in bicelles (Figure 10). As one can see in Figure 10, in the absence of tryptophan, photolysis is accompanied by significant decrease in the intensity of KP signals (by approximately 40% after 64 laser pulses in PBS and 30% in bicelles). At the same time, in the presence of tryptophan, there is practically no decrease in the intensity of KP signals, which allows us to suggest that a reversible reaction of KP with tryptophan is the main reaction channel. The high efficiency of quenching the excited state of KP by tryptophan may be related to their close interaction in the ground state, in the lipid bilayer. As was mentioned previously, this is indicated by a shift in the aromatic KP NMR signals in the presence of tryptophan, as well as by the data of the selective NOESY experiments.

It should be noted that the CIDNP pattern changes significantly with decreasing pH of the solution, namely, polarized reaction products appear in the region of 8–10 ppm (Figure 9). This range of chemical shifts is characteristic of lipid oxidation products [39]. Also, polarization of aromatic protons of KP or its reaction products are observed at 7.5–7.8 ppm. Taking into account the results of MD calculations, we can assume that the protonated form of KP penetrates deeper into the lipid bilayer. These reaction products do not accumulate upon prolonged irradiation of the reaction mixture, which indicates their photoinstability. Note that polarization in this range of chemical shifts was not observed in the CIDNP spectrum in the absence of tryptophan, which allows us to conclude that it is formed in secondary processes during the photoinduced interaction of the reaction products with Trp molecules. This is also evidenced by the observed increase in the CIDNP intensity of these products during photolysis, accompanied by a decrease in the KP concentration. The absence of the polarization of Trp protons can mean that this radical reaction is irreversible. This result allows us to conclude that the phototoxicity of KP can be caused not only by its radical reactions with lipids and embedded peptides, but also by photosensitive products of these reactions.

## 4. Conclusions

Thus, the study of photoinduced processes with participation of the nonsteroidal anti-inflammatory drug ketoprofen in the lipid bilayer showed a significant effect of the nature of the medium and the presence of potential electron donors on the mechanism of photolysis. Due to its lipophilicity, KP easily penetrates into the lipid membrane, but the depth of penetration into the membrane decreases for the deprotonated form of KP. If in a homogeneous solution the main channel of KP photolysis is monomolecular decomposition, then, in the presence of lipids, the equilibrium shifts towards the bimolecular reaction of KP photoreduction. This reaction results in irreversible damage of the lipid membrane due to the formation of oxidized products and the initiation of cyclic radical lipid polymerization. Using the amino acid tryptophan as an example, it was shown that, at comparable concentrations, the radical reaction of KP with tryptophan proceeds more efficiently than with lipid molecules. Selective NOESY NMR experiments showed the presence of binding between the molecules of KP and tryptophan in the ground state in the lipid membrane. Considering that natural cell membranes include a large number of peptides (ion channels, receptors, etc.), this result is important for understanding the possible mechanisms of photoinduced KP cytotoxicity. All of the above reaction channels (monomolecular decomposition, abstraction of a hydrogen atom from a lipid or amino acid molecules with the formation of a ketyl radical) lead to the formation of free radicals, sources of KP phototoxicity, as well as a wide range of photolysis products, sources of its photoallergicity. The reaction products of KP with lipids (in particular, aldehydes) are themselves photoactive and enter into further radical photochemical reactions, leading to further damage to the membrane.

## Figures and Tables

**Table 1:** Spin-lattice (T_1_) relaxation times of ketoprofen signals (chemical shift 7.8 ppm) and tryptophan protons (chemical shift 7.2 ppm) in different media.

	Water, pH = 7	Bicelles, pH = 7	Bicelles, pH = 3.5
KP (7.8 ppm) without Trp	2.5 ± 0.2 s	1.2 ± 0.1 s	1.1 ± 0.1 s
KP (7.8 ppm) with Trp	3.1 ± 0.1 s	3.0 ± 0.1 s	1.0 ± 0.2 s
Trp (7.2 ppm)	2.4 ± 0.2 s	2.6 ± 0.1 s	1.9 ± 0.2 s

## Data Availability

Data is provided in the article.

## Abbreviations

CIDNP	chemically induced dynamic nuclear polarization
DHPC	1,2-diheptanoyl-sn-glycero-3-phosphocholine
DMPC	1,2-dimyristoyl-sn-glycero-3-phosphocholine
KP	ketoprofen
MD	molecular dynamics
NMR	nuclear magnetic resonance
NOESY	Nuclear Overhauser effect spectroscopy
PBS	phosphate buffered saline
PME	particle mesh Evald
SPC	single point charge
Trp	tryptophan
CIDNP	chemically induced dynamic nuclear polarization
